# Molecular Biomarkers for Celiac Disease: Past, Present and Future

**DOI:** 10.3390/ijms21228528

**Published:** 2020-11-12

**Authors:** Aarón D. Ramírez-Sánchez, Ineke L. Tan, B.C. Gonera-de Jong, Marijn C. Visschedijk, Iris Jonkers, Sebo Withoff

**Affiliations:** 1 Department of Genetics, University of Groningen, University Medical Center Groningen, 9700 RB Groningen, The Netherlands; a.d.ramirez.sanchez@umcg.nl (A.D.R.-S.); i.l.tan@umcg.nl (I.L.T.); i.h.jonkers@umcg.nl (I.J.); 2 Department of Gastroenterology and Hepatology, University of Groningen, University Medical Center Groningen, 9700 RB Groningen, The Netherlands; m.c.visschedijk@umcg.nl; 3 Department of Pediatrics, Wilhelmina Hospital Assen, 9401 RK Assen, The Netherlands; Gieneke.Gonera@wza.nl

**Keywords:** celiac disease, new biomarkers, diagnosis, follow-up, non-invasive

## Abstract

Celiac disease (CeD) is a complex immune-mediated disorder that is triggered by dietary gluten in genetically predisposed individuals. CeD is characterized by inflammation and villous atrophy of the small intestine, which can lead to gastrointestinal complaints, malnutrition, and malignancies. Currently, diagnosis of CeD relies on serology (antibodies against transglutaminase and endomysium) and small-intestinal biopsies. Since small-intestinal biopsies require invasive upper-endoscopy, and serology cannot predict CeD in an early stage or be used for monitoring disease after initiation of a gluten-free diet, the search for non-invasive biomarkers is ongoing. Here, we summarize current and up-and-coming non-invasive biomarkers that may be able to predict, diagnose, and monitor the progression of CeD. We further discuss how current and emerging techniques, such as (single-cell) transcriptomics and genomics, can be used to uncover the pathophysiology of CeD and identify non-invasive biomarkers.

## 1. Introduction

Celiac disease (CeD) is a complex immune-mediated disorder triggered by dietary gluten in genetically predisposed individuals. The estimated worldwide prevalence of CeD is very high (1–1.5%) [1]. The disease is characterized by inflammation and villous atrophy of the small intestine that can lead to gastrointestinal complaints, malnutrition, and malignancies. The clinical spectrum of CeD is, however, broad and can include extra-intestinal symptoms, such as anemia, fatigue and dermatitis herpetiformis [2]. These factors make CeD complicated to diagnose, and it is estimated that only 1/3 to 1/9 of all CeD patients are properly diagnosed [3]. Once diagnosed, the only available treatment for CeD is a strict life-long, gluten-free diet (GFD).

The most recent guidelines recommend starting the diagnostic process for CeD in (1) patients with symptoms suggestive of CeD, (2) individuals with laboratory abnormalities previously associated with CeD (e.g., those indicative of malabsorption), or (3) other risk groups such as first-degree family members of CeD patients, patients with Type I Diabetes Mellitus, and patients with Down Syndrome [4,5]. Concerning these recent recommendations, serological testing for the presence of antibodies against gliadin and deamidated gliadin peptide has been replaced by testing for Immunoglobulin A (IgA) antibodies against tissue transglutaminase (anti-TG2) and endomysium (anti-EMA), which both display higher sensitivity and specificity [4]. The diagnostic procedure also differs between adults and children. Regarding adults, serology is combined with histopathological evaluation of small-intestinal biopsies [4], and it is important to include a duodenal bulb biopsy to increase the diagnostic yield [6]. CeD disease status is subsequently scored based on decreased villous height-to-crypt depth ratio (villous atrophy and crypt hyperplasia) and the influx of intraepithelial lymphocytes (IELs). The histological classification used for CeD is called the Marsh classification [7]. Regarding children, high antibody titers (anti-TG2 ≥ 10 times the upper limit of normal and positive anti-EMA in a second blood sample) are sufficient to establish CeD, which makes the diagnosis less invasive by eliminating the need for duodenal-endoscopy [5]. Children with anti-TG2 levels < 10 times the upper limit of normal still require a biopsy to confirm CeD.

Since more than 99% of CeD patients are human leukocyte antigen (HLA)-DQ2- or HLA-DQ8-positive, HLA-genotyping does not have added value for diagnostic purposes, since positive serology correlates almost perfectly with the presence of these HLA-types [8]. Moreover, these HLA haplotypes also occur in the general population with a frequency of approximately 40% [9,10]. However, due to its requirement for the development of CeD, HLA-genotyping can be used to rule out CeD, for example in first-degree relatives of CeD patients or in individuals who self-diagnose with CeD and start a GFD [4,5].

During this review, we summarize the strategies that are used to identify potential novel molecular biomarkers for CeD diagnosis and follow-up. To enable an understanding of how diagnostic CeD biomarkers might be involved in CeD etiology, we first summarize the immunopathology of CeD.

## 2. CeD Immunopathology

**Figure 1:**
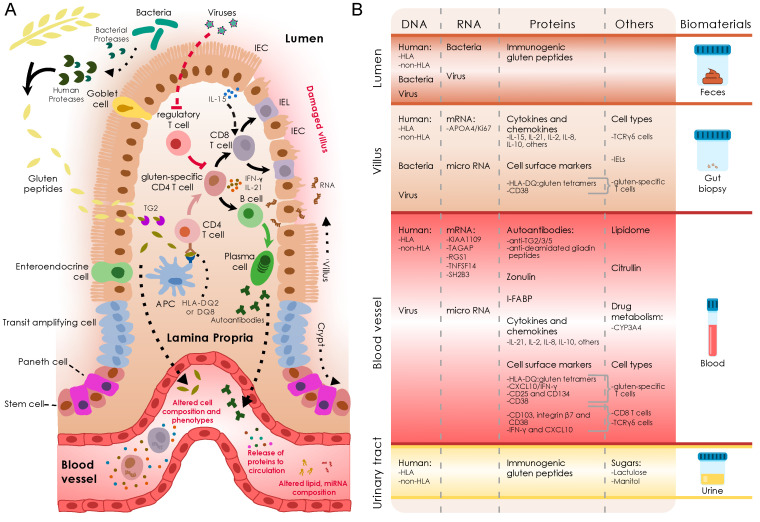
(**A**) Schematic representation of the immunopathology of Celiac Disease (CeD). Dietary gluten is partially degraded by human and microbial proteases. These peptides pass the epithelial layer (IEC: Intestinal epithelial cell) by paracellular or transcellular transport. Upon entering, tissue transglutaminase 2 (TG2) deamidates the gluten peptides, which are then processed by antigen presenting cells (APCs) and presented to CD4+ T cells in the context of human leukocyte antigen (HLA)-DQ2 or HLA-DQ8. After a process of selection, gluten-specific CD4+ T cells propagate and orchestrate the immune response by producing specific cytokines such as interleukin (IL)-21 and interferon-gamma (IFN-γ). Combined with IL-15, these cytokines promote the development of B cells into antibody-producing plasma cells and the activation of intraepithelial lymphocytes (IELs), which acquire cytotoxic properties to attack intestinal epithelial cells, thereby causing villus atrophy. The immune response in CeD causes modifications observable in blood such as release of immune- or damage-related markers (highlighted in red). Figure adapted from Moerkens and Mooiweer et al., [35]. (**B**) CeD biomarkers currently under study categorized by different compartments (rows, left) and separated by biotype (columns). Biomarkers that can be analyzed in easily collected biomaterials rather than invasive biopsies are more desired for diagnostics (rows, right).

As a complex disease, the pathophysiology of CeD involves a combination of environmental, genetic, and immunological factors, and possibly also microbial factors (Figure 1).

The most important environmental factor involved in CeD is gluten exposure [11,12]. Glutens are storage proteins commonly found in grains that are widely used in the Western diet (wheat, barley, and rye). Due to their high proline content, gluten proteins are inefficiently degraded into peptides in the small intestine, where they traverse the intestinal barrier and reach the lamina propria by transcellular or paracellular routes (Figure 1A) [13]. Regarding the lamina propria, gliadin peptides are deamidated by tissue transglutaminase 2 (TG2) and can be taken up by antigen-presenting cells. Specific deamidated gliadin molecules have a very high affinity for the CeD-associated HLA-DQ2 and -DQ8 molecules, which leads to the generation and activation of a pool of gluten-specific CD4+ T cells [14,15,16]. Activated gluten-specific T cells then respond and produce the pro-inflammatory cytokines interleukin (IL)-21 and interferon-gamma (IFN-γ) [17]. Furthermore, IL-15, which is a hallmark for CeD, is upregulated in both the epithelial barrier and the lamina propria. Together, these cytokines orchestrate a cascade that leads to increased stress in intestinal epithelial cells, migration of CD8+ cells to the epithelial layer, and activation of B cells. Upon arrival in the epithelial layer, the CD8+ T cells (designated CD8+ intraepithelial lymphocytes (IELs)) are effectively “licensed to kill” epithelial cells, thereby damaging the villous structure of the small intestine (villous atrophy) [17]. Gluten-specific T cells also promote the activation of B cells, which develop into plasma cells, thereby producing the auto-antibodies that are used as the biomarkers in CeD serology tests (Figure 1A) [4,18]. Moreover, a recently proposed hypothesis suggests that B cells may have an antigen-presenting role in CeD [18,19].

Although gluten peptides play a critical role in CeD immunopathology, not all CeD patients develop CeD upon their first intake of gluten. It has been postulated that viral (e.g., reovirus and norovirus [20,21]) and/or fungal infections (Candida [22]) create the environment for an anti-gluten response to be elicited. Bacterial microbiota also has been implicated in CeD but may act in a different way than viruses. Duodenal microorganisms have different capacities to degrade gluten. Pseudomonas aeruginosa, a bacteria enriched in the duodenum of CeD patients for example, produces elastases that can degrade and modify the gluten to produce gliadin peptides that are highly immunogenic [23], which may explain how the microbiota can cause imbalance in the immune homeostasis (Figure 1A). Additionally, it has been shown that some bacterial protein fragments can elicit a stronger activation of gluten-specific T cells by binding to HLA-DQ2 [24,25]. However, there is currently no evidence for which microbes might produce these peptides in CeD patients.

Genetics plays a pivotal role in CeD. The HLA-DQ2 and/or -DQ8 haplotypes are required to mount the specific response against gliadin peptides. However, ~40% of the Western population are DQ2/DQ8 carriers, even though only 3% of DQ2/DQ8 carriers develop CeD [10,26,27]. DQ2/DQ8 carriership, thus, is essential for the development of CeD, but carriership is not the cause. Thus far, genome-wide association studies have identified more than 40 non-HLA risk loci associated with CeD [28,29]. Together, these loci and the HLA loci explain more than 40% of the heritability of CeD [30]. Many of the genes in these loci are immune genes [31]. Since most of the non-HLA CeD single-nucleotide polymorphisms (SNPs) are located in the non-coding genome, they are likely to contribute to CeD pathology by affecting the expression of genes involved in the biological pathways that are perturbed in CeD. Although a single SNP might affect only the risk of developing CeD to a small extent, a combination of multiple SNPs and loci may affect downstream central hub genes that could implicate novel biomarkers and therapeutic targets [31,32]. Accompanying the recent publication of data from large case–control genome-wide association studies and population controls, such as the UK biobank, is the now possible ability to calculate genetic risk scores that combine the additive risk of multiple CeD risk-SNPs into one score to indicate the risk of developing CeD [10,33,34]. Indeed, a genetic risk score based on only 46 SNPs differed significantly between CeD patients and controls, which makes genetic risk scores easier to interpret and implement in future clinical applications than the whole-genome panels that are used in case–control studies [34].

It is a challenge to clearly identify the individual contributions of environmental, immunological, and genetic factors to CeD, as none of the currently known factors are sufficient to explain CeD risk completely. It seems that all these factors are part of an interconnected puzzle that causes loss of tolerance to gluten and the subsequent clinical manifestations of CeD. Fundamental studies can help to unravel CeD pathogenesis and identify key players and biomarkers for early detection, monitoring and control of CeD.

## 3. Novel Developments in Diagnosis

Rapid screening of high-risk populations is expected to decrease the number of undetected CeD cases [36]. Recently, effort has been invested in developing point-of-care tests that allow for quick, non-invasive, cost-effective, user-friendly diagnosis, for instance a test based on detection of IgA anti-TG2 antibodies in finger prick blood [37,38]. Unfortunately, the clinical studies performed thus far show that the accuracy and, especially, the sensitivity of these tests need to be optimized before widespread implementation [4,5,37,39].

Novel developments in endoscopic techniques now can capture the location and extent of villous atrophy more directly. These approaches include chromoendoscopy (mucosal staining with a specific dye), confocal endomicroscopy (microscopic visualization during upper-endoscopy) and non-invasive techniques such as capsule endoscopy, and they have been reviewed extensively elsewhere [40].

## 4. Why Do We Need Novel Biomarkers?

To date, CeD diagnosis relies on serology and on biopsies acquired in an invasive manner. Despite diagnostic advances, there are several reasons to keep searching for additional novel biomarkers to improve CeD diagnostics and follow-up.

(1) Serological tests can lead to false-negative or false-positive results.

Current serological tests can yield false negatives in IgA-deficient patients. About 2–3% of all CeD patients display IgA-deficiency, a 10-fold higher incidence than in the general population [41]. False-positive results have been observed in several other (auto-)immune related diseases, such as primary biliary cholangitis, and in enteric infections [4,42,43]. Moreover, there is a patient group coined ‘potential CeD patients’ who have positive serology but no villous atrophy who, therefore, may not need to follow a GFD [44,45]. However, the estimated cumulative incidence of children with potential CeD who develop villous atrophy within the 12-year follow-up after first seropositivity is around 43% [45].

(2) Prevention of severe small-intestinal damage.

Anti-TG2 only appears in circulation after the villous structure of the small intestine is affected. Novel biomarkers that enable the detection of CeD-onset (for instance in high-risk individuals) could lead to rapid initiation of a GFD. This would prevent full-blown disease and could be helpful for distinguishing which potential CeD patients will progress to CeD and which will not.

(3) GFD adherence and response is difficult to monitor.

It is difficult to adhere a strict lifelong GFD. Added to the social consequences of a GFD, unintentional gluten intake is common when following a GFD due to cross-contact from various sources, including dietary supplements and even playdough [46]. Currently, GFD adherence is monitored by dietetic review and serology. However, unintentional gluten intake, and the challenges of monitoring gluten intake in young children, make it difficult to interpret dietetic reviews and the serological markers, and even the absence of clinical symptoms, correlate poorly with mucosal healing [47,48,49,50]. Regarding cases with persistent symptoms but without elevated anti-TG2, it would be useful to have additional biomarkers that could exclude dietary lapses as a cause of symptoms. Also, in the case of intentional gluten intake, for example during puberty, additional biomarkers possibly could reflect gluten intake with or without mucosal damage without elevated levels of anti-TG2. Novel tools that allow sensitive and rapid gluten monitoring, ideally by patients themselves, would help to avoid certain foods and behaviors.

(4) Comorbidities and complications.

Although the most severe complaints may improve within several weeks after starting a GFD, mucosal recovery is only achieved in about 50% of CeD patients after one year of GFD, even when a strict diet is followed [48,51]. The persistence of intestinal damage is associated with a higher rate of CeD-associated complications such as bone abnormalities and malignancies [48,51]. Currently, we have no biomarkers that predict the onset of co-morbidities in CeD patients, such as dermatitis herpetiformis, other immune-mediated diseases (e.g., Type I Diabetes or thyroid diseases), or severe complications such as refractory CeD or enteropathy-associated T cell lymphoma.

(5) Clinical trial evaluation for the development of new treatments.

Assessing villous damage in biopsies is currently the method of choice to evaluate treatment response to novel drugs for CeD; however, non-invasive markers for mucosal damage or infiltration of IELs would help in clinical trials to evaluate treatment response [52].

To summarize, the search for novel biomarkers is crucial to improving early diagnosis, decreasing diagnostic burden, testing treatment efficacy, and improving follow-up and monitoring of CeD comorbidities after the start of a GFD. Ideally, these biomarkers should be detectable in a material that can be obtained in a non-invasive or minimally invasive manner, such as blood, feces, or urine.

## 5. Non-Invasive and Minimally Invasive Biomarkers

Regarding the next sections, we discuss potential novel non-invasive and minimally invasive biomarkers for diagnosis and follow-up of CeD and give an overview of how state-of-the-art techniques could lead to a better understanding of CeD pathology and to novel biomarkers. An overview of the biomarkers discussed is presented in Figure 1B and Table 1.

### 5.1. Cytokines, Chemokines and Other Proteins Detectable in Blood

Cytokines and chemokines are key players in the immunopathology of CeD. It is important to realize that the key driving cytokines or chemokines that are involved in disease initiation, maintenance, and/or progression may not be detectable in blood. It is possible that these biomarkers are produced in narrow windows of time or they might be diluted to undetectable levels in circulation. Nevertheless, some of these proteins are relevant for diagnostics because they reveal specific signature changes in CeD that can highlight different stages of disease progression.

Basal levels of some cytokines are increased in patients with active CeD compared to patients on a GFD and using healthy controls. Previous reports describe that the increased serum levels of some cytokines (such as IL-4, IL-10, IL-1α, IL-1β, IL-8 and IL-21) seen in CeD patients are correlated with IgA anti-TG2 titers and villous atrophy, making them candidates for diagnostic biomarkers [53,54]. Remarkably, the levels of some cytokines, such as IL-8, remain high for a long time after initiation of a GFD. This may be linked to the long recovery time of the duodenum and may present a way to detect CeD in patients already on a GFD.

New techniques to perform targeted high- or medium-throughput proteomics, such as the Olink platform [55], now make it possible to measure multiple protein markers using a small volume of sample. Using these techniques, circulating IL-2, IL-8 and IL-17A were detected in blood within two or three hours after gluten challenge in CeD patients but not in individuals with self-reported gluten sensitivity [56,57], probably reflecting rapid activation of ‘primed’ gluten-specific T cells upon antigen exposure. Although the authors found that cytokine response varied broadly among patients, 19 of 26 CeD patients (73%) versus just one of 67 self-reported gluten sensitivity patients (1.5%) were confirmed as IL-2 responders upon gluten challenge [58]. Interestingly, the patients with the highest levels of cytokines in the bloodstream also displayed the most severe symptoms [56,57].

To conclude, using the rapid rise of cytokines as a biomarker upon gluten-challenge may reduce the duration of current diagnostics methods that rely on two- to six-week long gluten challenges for an accurate result from serology or biopsy tests [4]. Gluten-related cytokine responses also may be useful clinical biomarkers for assessing patient recovery after CeD-mediated villous atrophy.

### 5.2. Cellular Composition of the Peripheral Blood Mononuclear Cell (PBMC) Fraction and Gene and/or Protein Expression

Differences in the composition of the peripheral blood mononuclear cell (PBMC) compartment in blood, or alterations in the expression profiles of these cells, may provide biomarkers for CeD. Cell types that are highly specific for CeD, such as gluten-specific T cells, but rare or not present in healthy individuals are of special interest [59].

Gluten-specific T cells can be observed at very low counts in circulation, an issue that can be overcome by enriching and/or staining them with HLA-DQ:gluten tetramers (a complex of four subunits of HLA-DQ2 binding to a gluten peptide) and subsequent fluorescence-activated cell sorting (FACS). Detection of gluten-specific T cells in circulation is a proposed marker for CeD that can detect CeD after the start of a GFD [59,60].

The HLA-DQ: gluten tetramer method requires staining and sorting the cells (FACS), which is labor-intensive and difficult to implement on a large scale. Therefore, there have been efforts to find non-invasive proxies for the number of circulating gluten-specific T cells in CeD using easier and less expensive methods that can be implemented on a larger scale. This includes the combined measurement of certain plasma cytokines (like C-X-C Motif Chemokine Ligand 10 (CXCL10)/ IFN-γ) by ELISA and the detection of the presence of CD25/CD134 positive cells with enzyme-linked immunospot (ELISPOT) [61].

The detection of surface markers in CeD-associated cell types also can provide valuable supplementary information. Regarding gluten-specific T cells, Zühlke et al., demonstrated that the expression of CD38 can distinguish CeD patients on a GFD and indicate a re-exposure to gluten [62]. Phenotyping of surface cell markers of CD8+ and gamma-delta (γδ) T cells is also a good alternative for diagnosing CeD in individuals who are already on a GFD. López–Palacios et al., showed, after a short three-day gluten challenge, both cell types co-expressed CD103, integrin β7 and CD38 in 15 out of 15 CeD patients but only in one of 35 controls [61]. Even with the necessity of a laborious technique like FACS, these biomarkers may have potential for monitoring the efficacy of drugs to treat CeD without the need for an invasive biopsy.

RNA extracted from peripheral blood cells reflects the cellular composition and state of blood and, thus, may contain non-invasive markers for CeD diagnosis. Although RNA is sensitive to degradation, it is easier and cheaper to detect than proteins because this usually requires less input material and test accuracy does not rely on the specificity of antibodies. Concerning CeD patients, an increase in Tumor Necrosis Factor Ligand Superfamily 13B (TNFSF13B) messenger RNA (mRNA) levels and a decrease in TNF Receptor Superfamily Member 9 (TNFRSF9) mRNA levels in whole blood has been observed [63]. Remarkably, in a longitudinal cohort of high-risk individuals, five genes (KIAA1109, T Cell Activation RhoGTPase Activating Protein (TAGAP), Regulator of G Protein Signaling 1 (RGS1), TNFSF14, and SH2B Adaptor Protein 3 (SH2B3)) were overexpressed in PBMCs of CeD patients at least nine months before CeD diagnosis. Based on expression of these genes, it was possible to classify CeD cases and controls in 95.5% of patients (*n* = 22) [64]. These predictive markers may be helpful for identifying individuals at high risk of CeD earlier than current serological markers, which could prevent mucosal damage and symptoms because patients could initiate a GFD earlier.

To conclude, detection of CeD-specific cell types in circulation or recognition of specific markers at protein- and RNA-levels may allow for earlier diagnosis of CeD than current serological methods and allow for diagnosis without the need for a duodenal biopsy in individuals already following a GFD.

### 5.3. (Circulating) micro-RNAs

MicroRNAs (miRNAs) have been put forward as disease- or disease stage–specific biomarkers. MiRNAs are short RNAs (19–24 nucleotides) that play a role in post-transcriptional gene regulation [65]. The miRNA transcriptome can be disturbed in disease-affected tissues, and disease-specific differences have been measured in extracellular body fluids such as blood, saliva and urine [66,67,68].

Several miRNA studies have shown the potential of miRNAs as biomarkers for CeD. Studies on duodenal biopsies showed that the miRNA profiles of CeD patients differ significantly from those of controls [69,70,71,72,73,74,75]. Only a few studies are available on circulating miRNA profiles in plasma or serum samples [71,74,75,76], but there are indications that circulating miRNAs are differentially expressed between CeD cases and controls. MicroRNA-21 is upregulated in both duodenal biopsies and circulation, for example [71,74,75].

The function of extracellular miRNAs is under debate. It is feasible that miRNAs in circulation are a consequence of tissue damage, but it also has been suggested that miRNA-containing vesicles play a role in the immune synapse and they might act as “micro-hormones” and function elsewhere in the body [77,78]. This second hypothesis is supported by the findings that miRNAs are selectively packaged in extracellular vesicles and miRNAs secreted by a donor cell type can be taken up by other cells and regulate gene-expression [79,80,81]. Thus, future studies also should assess the advantages of using miRNAs as potential prognostic markers for CeD.

### 5.4. Microbiome and Virome

#### 5.4.1. Microbiome

Growing evidence supports the hypothesis that the gut microbiome plays an important role in CeD pathogenesis. Generally, it has been shown that “beneficial” microbes, such as some species of Bifidobacterium and Lactobacillus, are decreased in the duodenum of CeD patients, while pro-inflammatory bacteria, such as Proteobacteria, are more prevalent when compared to healthy individuals [82]. Olivares et al., showed that children at high risk of developing CeD (HLA-DQ2 carriers with a first-degree relative affected by CeD) exhibit a different fecal microbiome composition than low risk (non-HLA-DQ2/DQ8 carriers with a first-degree relative affected by CeD) and healthy individuals [83]. Analysis of stool samples from pre-diagnosis early timepoints in infants who later developed CeD (*n* = 10) and children who remained healthy (*n* = 10) suggested that the HLA-DQ2 haplotypes may alter the early trajectory of gut microbiota and influence the maturation of the immune system [84].

Although gut microbiome dysbiosis may have potential for prediction of CeD, multiple environmental factors such as diet, age, sex, and use of antibiotics and other drugs also can affect microbiome composition. Therefore, potential biomarkers from the microbiome need exploration in larger cohorts.

#### 5.4.2. Virome

Like bacteria, viruses may act as protectors or triggers in CeD development. Potentially protective viruses include rubella, Epstein–Barr virus, cytomegalovirus, and herpes simplex type 1 virus [85]. Viruses that have been associated negatively with CeD include reovirus, rotavirus, enterovirus, adenovirus, hepatitis C virus, hepatitis B virus, and some strains of Epstein–Barr virus and cytomegalovirus [86,87]. Remarkably, exposure to specific viruses, such as reo- and rotaviruses, early in life is associated with a higher risk for CeD, suggesting that previous infections with this virus may have triggered CeD onset in some patients [20,88,89].

Viruses may affect mechanisms involved in oral tolerance to dietary antigens. Oral tolerance is the state in which the immune system accepts the intake of innocuous antigens found in food without mounting a rejection response [88,90]. Bouziat et al., showed in a mouse model how reoviruses can induce T helper type 1-associated immunity toward dietary antigens, thereby causing loss of oral tolerance, in line with observations from experiments with noroviruses [20,21].

To conclude, the exploration of the gut microbiome and the virome in larger and longitudinal studies may help to identify markers for disease onset and progression of CeD.

### 5.5. Lipids and Lipid Processing Genes as Markers for CeD

Digestion and absorption of lipids in the small intestine is disturbed in CeD because the surface area of the small intestine is reduced due to villous atrophy [91]. Studying the circulating lipidome and other proxies of disturbed lipid uptake and metabolism, therefore, might provide interesting biomarker candidates for CeD.

Recently, two independent prospective and longitudinal studies in children at high risk for CeD reported that lipid profiles were significantly different in serum samples of participants who developed CeD during follow-up compared to the participants who did not develop CeD [92,93]. Changes in phosphatidylcholines were observed in CeD patients early in life, even before the introduction of dietary gluten. The authors postulated that these differences are independent of the degree of villous atrophy and suggested that unknown genetic factors could be the cause. To contrast, a previous longitudinal study reported that the lipid profile at four months of age did not differ between the children who did develop CeD and those who did not [94]. Thus, candidate lipid biomarkers need to be validated on a larger scale before they are clinically applicable.

Considering small-intestinal biopsies of patients with CeD, there is significant deregulation of key genes or proteins involved in lipid metabolism pathways [91,95,96,97]. These include Fatty Acid Binding Protein 2 (FABP2 or I-FABP) and Apolipoprotein A4 (APOA4), which are currently being studied as potential biomarkers for CeD. When damaged, intracellular I-FABP is released by small intestinal epithelial cells and can be detected in circulation. Plasma I-FABP has been shown to be increased in CeD patients compared to controls and correlates with the degree of villous atrophy [98,99]. Moreover, after a two-week gluten challenge in patients with CeD, I-FABP levels increased in 80% of participants (mean 1.8-fold increase) [100]. Although the exact specificity/sensitivity of I-FABP as a CeD biomarker fluctuates, in most studies I-FABP has high specificity but lower sensitivity [98,99]. However, because increased I-FABP levels are associated with a range of enteropathies, specificity is expected to be lower if controls with gastrointestinal complaints other than CeD are included in specificity studies. Thus far, anti-TG2 serology remains the more reliable diagnostic biomarker in the studies where I-FABP also is measured [98,99]. Nonetheless, I-FABP might be useful for avoiding diagnostic biopsies in patients who have elevated anti-TG2 levels but who do not fulfill the criteria for serological diagnosis (anti-TG2 level > 10 times the upper limit of normal) [98]. Future independent studies are necessary to validate the added value of I-FABP in CeD diagnostics and to assess whether it could be used in early prediction or follow-up of CeD.

### 5.6. Citrulline as a Marker for Mucosal Damage

Plasma citrulline is derived specifically from small-intestinal enterocytes [101]. During a recent study of 131 adult CeD patients, plasma citrulline levels exhibited a comparable specificity to plasma I-FABP and a higher sensitivity to detect villous atrophy, making this an interesting biomarker candidate for monitoring villous atrophy [99].

### 5.7. CYP3A4 Metabolization as a Marker for Mucosal Damage

Cytochrome P450 3A4 (CYP3A4), which is highly expressed in epithelial cells along the small-intestinal tract, is a member of the Cytochrome P450 enzyme family that metabolizes a range of commonly used drugs including simvastatin, a cholesterol synthesis inhibitor. CYP3A4 reduction or inhibition leads to a reduction in the metabolism of specific CYP3A4 substrates [102,103]. Considering biopsies of CeD patients, CYP3A4 is decreased [96,97,104,105], leading to a reduction in the metabolization of substrates [102,103]. Morón et al., showed, after oral simvastatin intake, the maximum serum level of simvastatin was significantly higher in active CeD (*n* = 18) compared to healthy controls (*n* = 11), and patients on a GFD (*n* = 25) had simvastatin levels comparable to healthy controls [103]. CYP3A4 metabolizing capacity, therefore, might be an interesting non-invasive proxy for villous atrophy, although this requires taking serum samples following administration of drugs metabolized by CYP3A4, making this method less suitable for children.

### 5.8. Intestinal Permeability Measurements as Proxy for Intestinal Barrier Function

Intestinal barrier function is impaired in CeD, leading to an increased permeability compared to controls, and there have been efforts to use these observations as a biomarker for CeD [106].

Zonulin is a protein that regulates tight-junctions and can disturb intestinal barrier function. It was proposed as a marker for intestinal barrier integrity and is a drug target in clinical trials for CeD (AT-1001, larazotide acetate) [107,108,109,110] (Available online: https://clinicaltrials.gov/ct2/show/NCT03569007). Some studies suggest that zonulin is indeed higher in serum of patients with CeD versus controls, but serum values do not change upon start of the GFD, making zonulin unsuitable for monitoring in follow-up [108,111,112]. The current zonulin detection method also has limitations, including fluctuations within the same individual in time, and the low specificity for zonulin of some commercially available ELISA kits [112,113,114].

Non-invasive tests for intestinal permeability, such as the lactulose–mannitol ratio measured in urine, are based on the principle that large sugars like lactulose cannot pass the intestinal barrier under normal conditions but can pass if the integrity of the barrier is affected. The results of these permeability tests have been shown to differ between CeD patients and controls [111]. However, the reliability of these tests has been shown to be variable and, therefore, are not recommended as clinical biomarkers for CeD [4,106,115,116]. Nonetheless, sugar-based permeability tests in urine are the only completely non-invasive tests available to measure intestinal permeability and, thus, remain valuable in fundamental studies.

### 5.9. Gluten Peptides as Biomarkers for GFD Adherence

Immunogenic gluten peptides are interesting markers to measure dietary compliance. ELISA-based tests that detect immunogenic gluten peptides in feces, serum, or urine are sensitive enough to detect small quantities of gluten in the diet [117,118,119,120]. Immunogenic gluten peptides can be detected frequently in the stool of patients on a GFD [119]. These studies indicate that unnoticed dietary lapses are common, even in patients who report strict GFD adherence. Furthermore, 70% of patients positive for gluten peptides tested negative for anti-TG2 IgA, which suggests that these dietary lapses are not detected when only measuring anti-TG2 IgA [117,119]. Immunogenic gluten peptides in feces can be detected approximately three days after a gluten challenge. Immunogenic gluten peptides in urine show up sooner, but also disappear more quickly, which suggests that measuring gluten in urine might be more useful for identifying which dietary products contain gluten [118]. Currently, clinical trials [NCT03462979 clinicaltrials.gov] are testing the use of point-of-care immunogenic gluten peptide tests at home.

### 5.10. Antibodies against Tissue Transglutaminases to Detect Skin and Neurological Manifestations of CeD

Serological antibodies against tissue transglutaminase 3 (TG3) and 6 (TG6) have been suggested as biomarker candidates for extra-intestinal manifestations of CeD. Rapid diagnosis of dermatitis herpetiformis (by anti-TG3) and of gluten-induced neurological manifestations such as ataxia (anti-TG6) would be valuable but need further investigation before being implemented in the clinical setting [4,121,122,123,124].

## 6. Duodenal Biopsies as Source for Novel Biomarkers

Although there is considerable interest in identifying CeD biomarkers that can be found in samples that can be collected in a non-invasive manner, it is also clear that the disease focus is on the small intestine. Due to this, fundamental research is focusing on small-intestinal samples of CeD patients and on the cell types present therein. Novel high-throughput techniques are currently being applied to uncover pathogenic pathways that are altered in the small intestine of CeD, including (single-cell) transcriptomics, medium and high-throughput proteomics, and cytometry by time-of-flight (CyTOF). It is hoped that these techniques open new avenues that lead to novel biomarkers for CeD diagnostics and monitoring.

### 6.1. Transcriptomic Studies: Markers for Small-Intestinal Damage

Transcriptome studies of intestinal biopsies of CeD patients have revealed genes and pathways that are altered by disease which, therefore, have potential as markers for small intestinal damage and function.

The transcriptome of the small-intestine can be used as a marker for the villous-to-crypt-ratio measured in histopathological slides [125,126]. The ratio of two genes, APOA4:Ki67, correlates well with the degree of villous atrophy, for example. APOA4 is a lipid-processing gene highly expressed in intestinal villi, whereas Ki67 is a broadly used cellular proliferation marker expressed in the intestinal crypts [69,95,104,127]. Measuring these genes in biopsies could help to reduce observer variation in reviewing histological slides and allow assessment of villous atrophy in (public) RNA-sequencing data for which the villous-to-crypt ratio is not available.

Added to the examples discussed above of how deregulated pathways in CeD, such as drug-metabolization, have led to biomarkers for CeD, there are other pathways/genes identified by transcriptome studies that might be worth exploring as non-invasive markers. Lactase (LCT) has a lower expression in CeD biopsies [95,97,104]. LCT encodes for the enzyme that breaks down lactose, and lactase activity can be measured reliably by a non-invasive hydrogen breath test [128]. Lactose malabsorption is common in CeD, and there are indications that this phenotype improves upon adopting a GFD [128,129]. Furthermore, among the upregulated immune-related genes in CeD biopsies, some can be measured in feces. These include the gene S100A9, which forms the heterodimer calprotectin, and the antimicrobial peptide lipocalin (LCN2, also known as NGAL (Neutrophil Gelatinase-Associated Lipocalin)), both of which are used as fecal biomarkers for disease activity in inflammatory bowel disease [104,116,130,131,132]. Deregulation of these genes is not specific for CeD but might be a potential proxy for small-intestine health, either individually or combined with other markers for mucosal damage.

### 6.2. Single Cells to Multi-Dimensions

The proteome or transcriptome profile of bulk samples, such as small-intestine biopsies or blood of CeD patients, is mainly driven by the cell type–composition of each tissue. However, the more abundant cells may overshadow the expression of rare cells present in tissues. Therefore, the use of high-throughput techniques, especially those that allow the characterization of single cells, is essential in the study of complex diseases.

Classically, FACS has been used to study cell surface markers and internal proteins in single cells. To date, FACS allows the analysis of up to 20 proteins at the same time in millions of cells. Recently, CyTOF has emerged as a technology that combines the principle of FACS and mass spectrometry. CyTOF allows the study of around 40 surface markers in millions of cells [133]. Recently, van Unen et al., applied this technology to gut biopsies and PBMCs of CeD patients, refractory CeD patients, and Crohn’s disease patients and pinpointed differences between the three patient groups [134].

Another emerging approach is single-cell RNA sequencing (scRNAseq) [135], which characterizes the transcriptome at a single-cell level. While the number of cells that can be analyzed simultaneously by scRNAseq is low compared to FACS and CyTOF (thousands to millions, respectively), the number of markers that can be analyzed increases to thousands of genes. ScRNAseq also does not require prior knowledge about which markers to use. Considering the context of CeD, Atlasy et al., identified an Natural Killer T–like cell subset that was absent in the duodenum from CeD patients and CeD-specific transcriptome changes in T cells, myeloid cells, and mast cells [136].

ScRNAseq also can be combined with methods that detect other layers of data in the same cell. Some examples of multilayer techniques include the characterization of cell surface markers (CITE-seq [137]), whole genome screening of open (active) chromatin (single-cell RNA/ATAC-seq [138]), actual position in the tissue by spatial transcriptome reconstruction, or mass cytometry imaging [139,140,141]. All these advances hold promise as the foundation for a multidimensional understanding of complex diseases. Henceforth, combining these high-throughput multi-omics studies with new model systems for CeD, like organ-on-chip technology [35], can help identify potential biomarkers in the pathogenesis of CeD.

## 7. Conclusions and Future Perspectives

During this review we have discussed non-invasive biomarker candidates that may complement current diagnostics and monitoring of CeD. Some of these markers are already being validated and/or implemented in the clinic. We also briefly highlighted how modern high-throughput techniques can help find new targets for diagnostics, monitoring, and drug development.

The current serological markers, TG2- and EMA-antibodies, are the cornerstone of the diagnosis due to their high specificity/sensitivity. However, simultaneously measuring additional markers for intestinal damage or function, such as citrulline or I-FABP, could identify cases with villous damage in future, thereby reducing or replacing the need for invasive biopsies in cases with borderline serology. Even if the individual biomarker candidates discussed here turn out to be more general markers of intestinal damage or inflammation, and nonspecific for CeD, efforts should be made to assess the diagnostic value of using combinations of these biomarkers for CeD.

Currently, there are no biomarkers to predict who will develop CeD. Genetics may provide part of the key. Genetic screening already has the potential to identify those individuals at highest risk for CeD, and new algorithms are being tested to increase the predictive power of genetic risk scores [34]. Future studies also should focus on whether genetic risk scores have added value over the use of serology alone, and whether genetic risk scoring would help to identify individuals who would benefit from serological screening for CeD at specific points in their lives. Many of the other markers described here such as the lipid profile and changes in circulating cell types or gene/protein expression, are also worth investigating as predictive tools for CeD.

Previously mentioned, one of the disadvantages of current diagnostics based on antibodies is that they cannot be used to diagnose patients already following a GFD. The option to establish the diagnosis in patients who are on a GFD after a single dose of gluten by measuring specific circulating cytokines is worth exploring because it eliminates the need for a longer gluten challenge, which may cause intestinal damage and symptoms. Measuring cytokines also could be a quick assay to assess the response to a gluten challenge after administration of adjuvant treatments in clinical trials. This would have huge benefits for assessing drug efficacy in CeD, since invasive duodenal biopsies would not be required to assess how patients are responding to a gluten challenge.

Hereafter, even dietary lapses and unintentional exposures might be detectable by measuring gluten peptides in urine, as studies so far have shown that these are quicker and more sensitive markers than anti-TG2.

Next steps would be to further explore the heterogeneity of CeD to identify markers that could help to predict who will develop complications associated with CeD or other immune-mediated diseases. We would like to emphasize that fundamental studies investigating the pathogenesis of CeD are essential in working toward more personalized diagnostics, monitoring, and treatment of CeD. Results of current and future fundamental research have yielded and will yield interesting non-invasive markers for CeD.

## Figures and Tables

**Table 1:** Overview of current and future biomarkers for celiac disease (CeD).

Based on Detection of:	Functional Group	Molecular Biomarker	Detectable in:	Comments	Practical Considerations
DNA	HLA-DQ2 or DQ8	HLA-DQ2 or DQ8	Virtually any human tissue	Useful in situations with expected false-negative serology. Negative HLA-DQ2/DQ8 excludes CeD reliably without need of gluten challenge. Positive test requires additional tests.	PCR-based tests available.
	Non-HLA loci	Risk variants	Virtually any human tissue	>40 risk loci identified, mostly in non-coding regions. Prognostic value of genomic risk scores need to be evaluated (who will develop CeD and who will not). Future studies: crucial to find associated genes and pathways to elucidate pathogenesis, potential new biomarkers and treatments.	Research in discovery phase. SNP based tests need to be developed if genomic risk score is proven to have sufficient diagnostic value.
Microbial DNA/RNA	Microbiome	Not yet available	Feces, brush biopsy	Enrichment for pro-inflammatory bacteria (Proteobacteria) and depletion of beneficial ones (Bifidobacterium and Lactobacillus). Studies necessary on the diagnostic/prognostic value of individual combined abundance of specific bacteria.	Research in preliminary discovery phase.
Viral DNA/RNA	Virome	Not yet available	Feces, brush biopsy, blood	Potential role in triggering the CeD by disturbing the oral tolerance. Associations found with CeD in reovirus, rotavirus, enterovirus, adenovirus, hepatitis C virus, hepatitis B virus, and some strains of Epstein-Barr virus and Cytomegalovirus. Studies necessary on the diagnostic/prognostic value of individual combined abundance of specific viruses.	Research in preliminary discovery phase.
RNA	Bulk mRNA	KIAA1109, TAGAP, RGS1, TNFSF14, and SH2B3	Blood	Transcripts overexpressed in RNA form PBMCs of CeD patients 9 months before diagnosis.	Potential use as predictor markers, further validation necessary before clinical application.
		APOA4:Ki67	Small intestinal biopsy	Biomarker for villous-to-crypt ratio in transcriptome data of biopsies, that eliminates observer variation in reviewing histological slides. Could help in basis (large-scale) transcriptome studies where no measured villous-to-crypt ratio is available and in clinical trials.	Requires small intestinal biopsy. Suitable to implement in clinical drug trials.
	Small non-coding RNAs	MicroRNAs	Small intestinal biopsy, blood	Differences detected between controls and CeD. Diagnostic and prognostic value to be determined.	Research in discovery phase.
Proteins	Antibodies	anti-TG2 IgA	Blood, saliva	Very high sensitivity/specificity for active CeD. Not reliable if individual is on GFD or has IgA deficiency. Less useful for follow up.	Currently used as a serological tool of choice in clinics. Saliva based and rapid on site point-of-care tests are under investigation.
		anti-TG2 IgG	Blood	IgG based tests (anti-TG2/anti-DGP) tests of choice in case of IgA-deficiency.	IgG based tests; have more inter-test variability than IgA-anti-TG2.
		Anti-Deamidated gliadin peptides (DGP) IgG	Blood	See IgG anti-TG2	See IgG anti-TG2.
		anti-EMA IgA	Blood	Used in combination with IgA anti-TG2 to confirm CeD in the non-biopsy approach.	Implemented in clinics. The indirect immunofluorescence test is more laborious and subjective than ELISA based anti-TG2.
		anti-TG3	Blood, skin biopsy	Diagnosis of Dermatitis Herpetiformis	Further validation is necessary before clinical applications.
		anti-TG6	Blood	Diagnosis of Gluten ataxia	Further validation is necessary before clinical applications.
	Cytokines and chemokines	IL-15	Small intestinal biopsy	Hallmark of CeD, involved in the T cell response. Elevated in CeD. Expressed on the surface of cells that are mainly located in gut.	Requires small intestinal biopsy.
		IL-21	Blood, small intestinal biopsy	Together with IL-15, involved in the T cell response. Elevated serum basal levels in CeD. Correlated with anti-TG2 titers.	Further validation is necessary before clinical application.
		IL-2	Blood, small-intestinal biopsy	Involved in the T cell response. Distinguishes CeD cases from self-reported gluten sensitivity patients. Increased within 2 h after gluten-challenge in CeD. Elevated serum titers is associated with worse symptoms. Distinguishes CeD cases from self-reported gluten sensitivity patients.	Further validation is necessary before clinical application. Requires a short (hours) gluten-challenge test.
		IL-8	Blood, small-intestinal biopsy	Involved in the T cell response. Elevated serum basal levels in CeD. Correlated with anti-TG2 titers. Increased within 2 h after gluten-challenge. Elevated serum titers are associated with worse symptoms. Takes more than one year of GFD to diminish to normal levels.	Further validation is necessary before clinical application. Can be used after a short gluten-challenge test, or as a long-term marker of recovery.
		IL-10	Blood, small-intestinal biopsy	Correlated with anti-TG2 titers. Elevated serum basal levels in CeD.	Further validation is necessary before clinical application. Requires a short (hours) gluten-challenge test.
		IL-17A	Blood, small-intestinal biopsy	Produced by T cells, mainly. Increased within 2 h after gluten-challenge. Elevated serum titers are associated with worse symptoms.	Further validation is necessary before clinical application. Requires a short (hours) gluten-challenge test.
		IL-1a	Blood	Elevated serum basal levels in CeD. Correlated with anti-TG2 titers.	Further validation is necessary before clinical application. Requires a short (hours) gluten-challenge test.
		IL-1b	Blood	Elevated serum basal levels in CeD. Correlated with anti-TG2 titers. Take more than one year of GFD to diminish to normal levels.	Further validation is necessary before clinical application. Potential use to assess the recovery of villus atrophy in long-term.
		IL-4	Blood	Elevated serum basal levels in CeD. Correlated with anti-TG2 titers.	Further validation is necessary before clinical application. Requires a short (hours) gluten-challenge test. Requires a short gluten-challenge test.
		Others	Blood	CCL20, IL-6, CXCL9, IFNγ, IL-10, IL-22, TNFα, CCL2, and amphiregulin.	Research in discovery phase.
	Peptides	Immunogenic gluten peptides	Urine, feces	Indicates presence of (unintended) gluten intake. Better marker for dietary adherence than IgA anti-TG2.	Can be detected in urine 3h after gluten intake, after 3 days in feces. Point-of-care at home tests are in clinical trials.
	Others	I-FABP	Blood	Non-invasive marker of villous atrophy. Indicates damage to small-intestinal enterocytes. Might be useful to identify patients that do not require additional biopsies to complement anti-TG2 if anti-TG2 is increased, but not >10x the upper limit of normal levels.	Note that elevated I-FABP is not specific to CeD, but occurs also in other enteropathies. Still, as a marker for intestinal damage is ready to be validated and implemented for clinical purposes.
		Zonulin	Blood	Marker for the intestinal barrier integrity.	Detectable by ELISA, but specificity and intra-individual fluctuations make it an unsuitable biomarker.
Cell-types	Gluten specific T-cells	HLA-DQ:gluten tetramers	Blood, small-intestinal biopsies	Complex used to identify gluten specific T cells by using their affinity to gluten epitopes.	Requires FACS, which is labor intensive, making it a less attractive biomarker for clinical applications.
		CXCL10, IFN-γ	Blood	Alternative to HLA-DQ:gluten tetramers to identify gluten specific T cells.	Uses ELISPOT, which is relatively easy to implement, but the test is not as specific as using tetramers.
		CD25, CD134	Blood	Alternative to HLA-DQ:gluten tetramers to identify gluten specific T cells.	Uses ELISPOT and FACS, which makes its use more difficult, thereby being less attractive in clinical applications.
		CD38	Blood, small-intestinal biopsies	Marker for subset of gluten specific T cells. Distinguish CeD on GFD patients. Capable of indicating a first exposure or a re-exposure to gluten.	Requires FACS, which is labor intensive, making it a less attractive biomarker for clinical applications.
	CD8 T cells	CD8	Blood	Relevant cells for CeD immunopathology, involved in the cellular mediated immunology.	Can be detected in blood by FACS after a short gluten challenge, being suitable candidates to diagnose CeD on GFD prospective patients.
	TCRγδ cells	TCRγδ	Blood, small-intestinal biopsies	Relevant cells for CeD immunopathology, used in the biopsy assessment. Cell count is highly increased in active CeD.	Requires FACS, which is labor intensive making it a less attractive biomarker for clinical application.
Metabolome	Lipidome	Not yet available	Blood	Lipid profile potential prognostic marker: Differences in lipidome detectable in a high risk cohort between children that will develop CeD versus those that will not, before the introduction of gluten. Might be useful to identify those patients that require intensive follow up with serology.	Research still in a preliminary, discovery phase.
	Amino acids	Citrulline	Blood	Non-invasive marker of villous atrophy. Amino acid specifically present in small-intestinal enterocytes. Circulating citrulline in blood is a proxy of small-intestinal enterocyte mass.	Note that elevated citrulline is not specific to CeD, but occurs in a range of diseases associated with small-intestinal damage. Still worthwhile to compare diagnostic yield with I-FABP, as citrulline might become a better predictor of villous atrophy.
	Drug metabolization	Metabolization rate drugs processed by CYP3A4	Blood	Non-invasive marker of villous atrophy. Indicates the expression of CYP3A4 in the small intestine and therefore a marker of presence of small intestinal epithelial damage.	Requires the administration of drugs. Grapefruit juice can influence the results. Likely not specific for CeD.
Sugars	Large sugars	Lactulose/Mannitol ratio	Urine	Indication of small-intestinal barrier function, different between CeD and controls.	Less attractive biomarker due to variation in the reliability of the tests. Still the only marker for intestinal integrity that can be measured non-invasively.

Abbreviations listed in the Table 1: human leukocyte antigen (HLA)); Polymerase chain reaction (PCR); single-nucleotide polymorphisms (SNP); peripheral blood mononuclear cells (PBMC); gluten-free diet (GFD); tissue transglutaminase (TG); endomysium (EMA); Immunoglobulin (Ig); interleukin (IL); fluorescence-activated cell sorting (FACS); Intestinal fatty-acid binding protein (I-FABP).
